# Combining Digital Cognitive Behavioral Therapy With Mindfulness Training for Binge Eating Disorder: Protocol for a Feasibility Trial

**DOI:** 10.2196/91761

**Published:** 2026-04-17

**Authors:** Margaret Sala, Corey Roos, Dante Ascarrunz, Casey M Stern, Jonathan Bricker, Jonathan M Feldman, Cheri Levinson, Mihaela Aslan, Jennifer L Hay, Hedy Kober

**Affiliations:** 1 Ferkauf Graduate School of Psychology Yeshiva University Bronx, NY United States; 2 Department of Psychiatry School of Medicine Yale University New Haven, CT United States; 3 Department of Pediatrics Albert Einstein College of Medicine Bronx, NY United States; 4 Division of Public Health Sciences Fred Hutch Cancer Center Seattle, WA United States; 5 Department of Psychiatry and Behavioral Sciences University of Washington Seattle, WA United States; 6 Department of Psychological and Brain Sciences University of Louisville Louisville, KY United States; 7 Department of Internal Medicine School of Medicine Yale University New Haven, CT United States; 8 VA Connecticut Healthcare System West Haven, CT United States; 9 Department of Psychiatry and Behavioral Sciences Memorial Sloan Kettering Cancer Center New York, NY United States; 10 Department of Psychology University of California, Berkeley Berkeley, CA United States

**Keywords:** cognitive behavioral therapy, mindfulness-based therapy, mindfulness-based intervention, eating disorder, binge eating disorder, digital

## Abstract

**Background:**

Cognitive behavioral therapy (CBT) is the most effective treatment for binge eating disorder (BED) but is limited by modest efficacy and low reach of in-person delivery. Combining mindfulness training with CBT could enhance efficacy by targeting emotion dysregulation, a key factor in BED that CBT does not adequately target. Digital delivery can enhance the reach of treatment. Accordingly, we developed *CBT-based Mindful Courage*, a 16-session digital intervention combining CBT and mindfulness training for BED.

**Objective:**

The aim of this study is to conduct a pilot randomized controlled trial to evaluate the feasibility (intervention and assessment completion) and acceptability (usability, overall satisfaction, engagement, visual appeal of content, understandability of program content, desire to continue the program, perceived skill acquisition, perceived confidence in implementing skills, and perceived helpfulness) of *CBT-based Mindful Courage*. We will also evaluate changes in outcomes (binge eating frequency, eating disorder symptoms, clinical impairment, and depression symptoms) and mechanisms (emotion dysregulation, dietary overrestriction, and trait mindfulness).

**Methods:**

This is a parallel-group, single-blinded, randomized controlled trial with a 1:1 allocation ratio. It will be conducted from November 2025 to November 2026, with study assessments at baseline, midtreatment, end-of-treatment, and 2-month follow-up. We will recruit a volunteer sample of adults with BED (N=40) aged 18 to 75 years via online advertisements. Exclusion criteria include a BMI <18.5, current anorexia or bulimia nervosa, and current treatment for BED or weight loss. Participants will be randomly allocated to an active control that includes coached self-monitoring (self-monitoring of food intake in *Recovery Record* and coaching calls by a research assistant) or to *CBT-based Mindful Courage+*coached self-monitoring. Both conditions will have a duration of 18 weeks. All study sessions will take place over Zoom.

**Results:**

The study was funded in April 2023. Data collection began in November 2025, and we anticipate recruitment to be completed by November 2026. Results are expected to be analyzed by May 2027 and published by May 2028.

**Conclusions:**

This will be an important first step in creating a scalable and efficacious treatment for BED.

**Trial Registration:**

ClinicalTrials.gov NCT07212673; https://clinicaltrials.gov/study/NCT07212673

**International Registered Report Identifier (IRRID):**

DERR1-10.2196/91761

## Introduction

Binge eating disorder (BED) is the most prevalent eating disorder (ED), affecting 17.3 million individuals worldwide [1]. Individuals with BED are at increased risk of several psychosocial and medical consequences [2-4] and elevated mortality [5]. Furthermore, BED is linked with high comorbidity [6] and reduced quality of life [7].

Conceptualizations of BED and other EDs have consistently focused on the notion that overrestriction (ie, excessive rigid restrictions in food intake) is a primary contributor to BED [8,9]. Consequently, cognitive behavioral therapy (CBT), the gold standard treatment for BED [10], has several behavioral components that directly target overrestriction [9].

However, CBT for BED has modest efficacy. Notably, approximately 50% of individuals who receive CBT for BED continue to report difficulties with binge eating after treatment [11]. The modest efficacy of CBT may be due to an inadequate focus on emotion dysregulation. Emotion dysregulation has been identified as a major contributing factor in the onset and maintenance of BED [12-14] but receives minimal attention in CBT treatments for BED. CBT interventions lead to only slight improvements in emotion dysregulation [8,15].

Mindfulness-based interventions (MBIs) are particularly effective for ameliorating emotion dysregulation [16,17] because they improve the ability to process and respond effectively to emotions. MBIs directly teach individuals to respond to emotions with awareness rather than automatically engaging in maladaptive emotion regulation strategies [18]. As such, they may help individuals with BED to accept emotions rather than react to them habitually (eg, by binge eating). Therefore, mindfulness training, the core component of MBIs, could be a promising addition to CBT for BED.

A few studies to date have investigated the use of MBIs in BED and produced preliminary evidence supporting the use of MBIs in the treatment of BED [19,20]. In addition, a recent review suggests that several MBIs for EDs result in significant reductions in emotion dysregulation [21]. Furthermore, our preliminary work indicates that MBIs result in large and significant decreases in emotion dysregulation in other EDs [22,23].

Integrating CBT and mindfulness training could lead to a potent intervention that targets two core mechanisms in BED: (1) excessive dietary restriction and (2) emotion dysregulation. Furthermore, mindfulness training may be synergistic with CBT. For example, mindfulness training may increase adherence to CBT skills by increasing the ability to tolerate emotional discomfort related to deploying CBT skills [24]. Similarly, CBT may enhance the ability to engage with the present moment and increase awareness by reducing focus on eating, weight, and shape.

Despite the promise of CBT and MBIs in the treatment of BED, a recent systematic review showed that only 20% of individuals in the community with a diagnosable ED can access ED-specific treatment [25] due to barriers such as a lack of experienced therapists, high cost of treatment, lack of health insurance, and shame and stigma associated with EDs and mental health treatment [26]. One way to overcome these barriers to treatment is to offer a digital treatment, that is, a multimedia application that can be delivered via smartphone or web. Digital MBIs and CBTs were already shown to be efficacious for several other psychiatric disorders [27-30]. While digital delivery of CBT has been shown to be efficacious for EDs [31], there are no digital MBIs to date for EDs outside of the ones developed in our preliminary work [22,23,32]. A recent meta-analysis of digital interventions for EDs included 8 treatments (4 for BED), and all interventions were based on CBT, with no interventions including mindfulness training [31]. Furthermore, the interventions included in this meta-analysis had various limitations, including high dropout rates, lack of engagement of the end user in the development stage, and modest effect sizes [31].

We developed a digital treatment combining CBT and mindfulness training for BED, called *CBT-based Mindful Courage.* We will conduct a small-scale randomized controlled trial (RCT) to assess the feasibility and acceptability of *CBT-based Mindful Courage*. The primary goals are to (1) evaluate feasibility, including intervention and assessment completion; and (2) evaluate acceptability, including dimensions of usability, overall satisfaction, engagement, visual appeal of content, understandability of program content, desire to continue the program, perceived skill acquisition, perceived confidence in implementing skills, and perceived helpfulness. Notably, the study is funded by the National Center for Complementary and Integrative Health (NCCIH) and guided by the NCCIH research framework [33] and the National Institutes of Health stage model for intervention development [34]. Accordingly, we will not conduct between-group analyses at this stage of development. Instead, we will evaluate within-treatment-condition mean changes in outcomes (binge eating frequency, ED symptoms, clinical impairment, and depression) and mechanisms (emotion dysregulation, dietary overrestriction, and trait mindfulness) as exploratory objectives.

## Methods

### Participant Recruitment and Eligibility Criteria

Participants will be recruited throughout the United States using advertisements posted on social media. The target sample size is 40 (n=20 in each treatment arm). This sample size was based on balancing various considerations, including ethical considerations (eg, participants’ time), resource use, prior feasibility trials [35], and availability of funding. Participant eligibility criteria are shown in Textbox 1.

**Participant eligibility criteria.**

**Inclusion criteria**

- Aged 18 to 75 years
- Ability to speak English fluently
- Meeting *Diagnostic and Statistical Manual of Mental Disorders, 5th edition* criteria for current binge eating disorder (BED)
- Willing and able to commit to the entire study protocol

**Exclusion criteria**

- BMI <18.5
- Requiring immediate treatment for medical complications
- Having current anorexia or bulimia nervosa or purging behaviors within the past year
- Being pregnant or breastfeeding
- Experiencing other severe psychopathology or medical illness that would limit the participants’ ability to comply with the demands of this study
- Currently receiving BED or weight loss treatment (treatment for other conditions will be allowed, as long as the treatment is not mindfulness based)
- Currently taking medications for weight loss or beginning medications that affect eating or weight within the last 6 months

### Study Design

This study is a 2-arm pilot feasibility RCT with parallel assignment and a 1:1 allocation ratio. All procedures reported here are derived from protocol version 1.0. Participants will be randomized to one of the following groups: (1) coached self-monitoring or (2) *CBT-based Mindful Courage*+coached self-monitoring. Participants will be blinded as to whether they received the active intervention or control condition. Outcome assessors and data analysts will be blinded to group allocation. All recruitment and study activities will be conducted online; the principal investigator and study staff will be located at Yeshiva University. Participants will complete interview-based and self-report assessments assessed at baseline, midtreatment, end-of-treatment, and 2-month follow-up on Health Insurance Portability and Accountability Act (HIPAA)–secured Zoom.

### Ethical Considerations

#### Ethics and Participant Safety

This study has received approval from the institutional review board at Yeshiva University (43318060.0). To our knowledge, the intervention does not pose any significant risks to participants, although some participants may become distressed while engaging in the treatment because we are addressing sensitive content (eg, difficult emotions, ED urges, and eating). All questionnaires have been validated in numerous prior studies. However, some questionnaires involve questions on ED symptoms and diagnoses and other psychological difficulties, which may elicit distress in some participants. All participants will provide signed consent after being informed of study procedures and objectives, possible risks, and the right to withdraw from participation at any time.

#### Compensation

Participants will receive up to US $180 for completing study assessments. This will include US $20 for the baseline visit, US $30 for the midtreatment assessments, US $40 for the end-of-study assessments, US $60 for the 2-month follow-up assessments, and US $30 for completing all lessons (for participants in the *Mindful Courage*+coached self-monitoring condition only). We decided to provide incentivization for app adherence because we are in an early stage of treatment development, and we wanted to ensure that participants were fully exposed to the intervention to provide acceptability ratings.

#### Data Privacy and Security

All data will be collected and stored via Qualtrics. Qualtrics is HIPAA secured and uses industry-standard encryption, supports access controls, and has audit logs. Deidentified data will be stored on secure cloud servers and will be retained for a minimum of 5 years. Full access to data will be given only to the principal investigator and approved research staff. Zoom sessions will not be recorded.

### Overview of CBT-Based Mindful Courage

#### Core Lessons

The digital intervention *CBT-based Mindful Courage* was developed through an iterative process considering patient feedback and consists of 16 sequential core lessons, which are completed across 18 weeks (Multimedia Appendix 1). Each lesson takes approximately 40 to 50 minutes to complete and includes several key features to promote engagement [36-39], including multiple animated videos, audio-guided meditation practices, interactive activities and questions to help participants understand the intervention content in a personally relevant manner, and supportive feedback. After completing each core lesson, participants are instructed to complete 2 audio-guided mindfulness meditation sessions that last 10 to 15 minutes before the next core lesson. The program includes 2 narrators, both clinical psychologists, who appear as avatars in the animated videos to guide participants through the lessons. The program also features several fictional patients, voiced by professional actors hired through Fiverr, who share their experiences through client testimonials and discussions with the 2 psychologists.

The CBT content is derived from enhanced CBT for EDs [9] and a condensed CBT for EDs [40]. The mindfulness content is adapted from a digital MBI for substance use disorder (NCT05852015) developed by Roos et al [35], a prior digital MBI for anorexia nervosa developed by Sala et al [22,23], and several standardized MBI and acceptance and commitment therapy protocols [41-45], particularly mindfulness-based relapse prevention [41].

The intervention also combines CBT and mindfulness techniques. For example, (1) we combine self-monitoring and mindful awareness to increase awareness of eating patterns that may trigger binge eating, (2) we encourage mindful awareness of the consequences of irregular eating, (3) we instruct participants to use mindfulness strategies to notice in their daily life how using CBT strategies (such as reducing dietary restraint and eating satiating meals) affects hunger or fullness and binge eating urges, (4) we combine reduction of valuation of weight and shape with values work, (5) we encourage awareness of ED behaviors and of the relationships between ED behaviors and ED symptoms, and (6) we encourage awareness of factors that may contribute to lapses and relapses.

#### Animated Content

**Figure 1:**
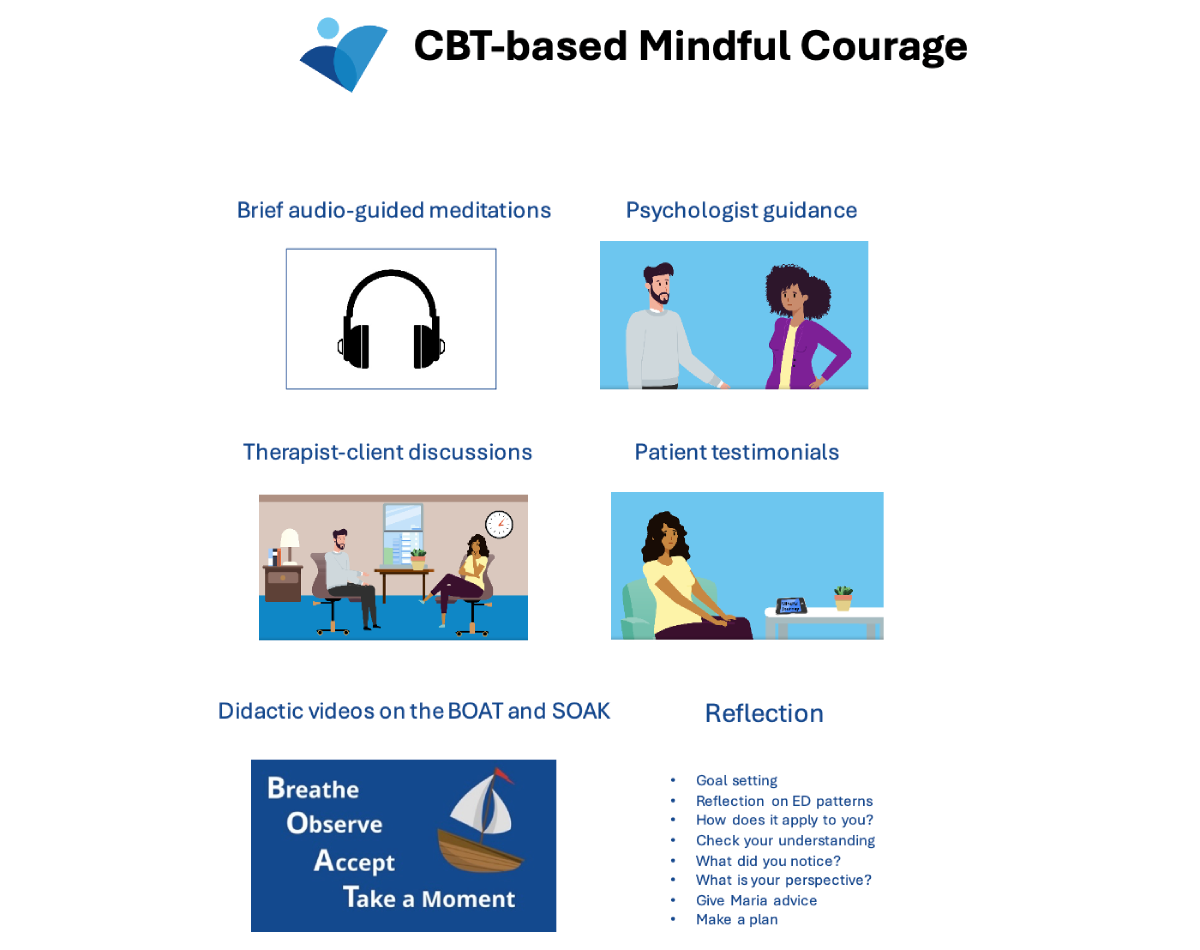
Example animations and video clips of cognitive behavioral therapy (CBT)–based Mindful Courage. BOAT: Breathe, Observe, Accept, Take a Moment; ED: eating disorder; SOAK: Stop, Observe, Appreciate, Keep Curious.

The animated videos were built with Powtoon, an online animation creation program; the digital lessons were created in Qualtrics; and videos were hosted in Vimeo. Audio content was recorded and edited with Audacity. See Figure 1 for examples of *CBT-based Mindful Courage* visuals.

### Overview of Coached Self-Monitoring

#### Recovery Record

Participants in both conditions will be asked to self-monitor their food intake using *Recovery Record* [46], a frequently used digital intervention among individuals with BED [47]. Participants will not be asked to engage with any other features of *Recovery Record.* We chose to ask participants from both conditions to track their food intake as regular eating is the foundation of ED recovery [48].

#### Phone Coaching

Participants in both conditions will receive phone coaching by psychology students during the 18-week treatment period but not during the follow-up period. Phone coaching for both conditions will include weekly coaching calls (10-15 minutes, except for week 1, which will be 30 minutes) and extra technical support as needed via phone and SMS text message. In addition, for participants in *CBT-based Mindful Courage*, there will be 1 to 2 weekly SMS text message reminders to use *CBT-based Mindful Courage* and additional phone or SMS text message contact (up to 1 weekly call and 2 weekly SMS text messages) for participants not engaging in *CBT-based Mindful Courage*. The aim of the phone coaching is to provide general motivational and technical support rather than provide any specific therapeutic intervention.

### Treatment Conditions

#### Coached Self-Monitoring

Participants in this condition will not have access to *CBT-based Mindful Courage* but will be asked to self-monitor their food intake in *Recovery Record* and will receive phone coaching.

#### CBT-Based Mindful Courage+Coached Self-Monitoring

Participants in this condition will have access to *CBT-based Mindful Courage* for 18 weeks. Participants will also be encouraged to engage with *Recovery Record* self-monitoring of food intake and will also receive phone coaching.

### Measures

Multimedia Appendix 2 [49-61] provides a summary of assessments and measures.

#### Screening Measures

We will establish BED diagnosis using the Structured Clinical Interview for *Diagnostic and Statistical Manual of Mental Disorders, 5th edition* [49]. Other inclusion criteria will be assessed with 1-item questions in an interview-based assessment.

#### Feasibility Benchmarks

Benchmarks for feasibility are based on previous digital interventions for EDs [31,62-64], and include (1) feasibility of recruitment (40 participants randomized); (2) feasibility of randomization (75% of eligible screens randomized); (3) intervention completion (34/40, 85% of randomized individuals completing at least 1 module; average completion rates of 60% for the 16 lessons) assessed objectively via Qualtrics; (4) completion rates for midtreatment, end-of-treatment, and 2-month follow-up visits (32/40, 80% of participants completing the midtreatment visit, 30/40, 75% of participants completing the end-of-treatment visit, and 26/40, 65% of participants completing the follow-up visit).

#### Acceptability Benchmarks

Acceptability will include dimensions of usability from the System Usability Scale [65]. We also created additional single-item questions, drawing upon the Technology Acceptance Model [66], evaluating overall satisfaction, engagement, visual appeal of content, understandability of program content, desire to continue the program, perceived skill acquisition, perceived confidence in implementing skills, and perceived helpfulness of *CBT-based Mindful Courage*. Participants in the *CBT-based Mindful Courage*+coached self-monitoring condition will rate their responses to each item from 1 (*strongly disagree*) to 5 (*strongly agree*). Given that this is the first pilot feasibility trial of *CBT-based Mindful Courage*, and our primary intention is to evaluate whether this intervention has preliminary promise for acceptability, benchmarks for these items will be conservatively set to an average score of >3.5 (*agree)*.

#### ED-Related Outcomes

BED-related outcomes, examined on an exploratory basis to estimate effect sizes for a full-scale RCT, will include (1) frequency of binge eating episodes in the last 28 days, which will be assessed with the Eating Disorder Examination Questionnaire (EDE-Q) [50]; and (2) ED symptoms, assessed with the global EDE-Q score. Additional outcomes will include (3) changes in clinical impairment measured via the Clinical Impairment Assessment [51] and (4) depressive symptoms measured via the 9-item Patient Health Questionnaire-9 [52]. All these measures have demonstrated adequate psychometric properties [51,67-72].

#### Mechanisms

We will measure mechanisms of change due to the intervention on an exploratory basis. This will include (1) emotion dysregulation, measured via the Difficulties in Emotion Regulation Scale-Short Form [53]; (2) dietary overrestriction, measured via the restraint subscale of the EDE-Q [50]; and (3) trait mindfulness, measured via the Cognitive and Affective Mindfulness Scale-Revised [54]. All these measures have demonstrated adequate psychometric properties [54,67,73].

#### Additional Measures

We will collect additional measures to be used in exploratory analyses. At baseline, we will collect data on duration of illness, past treatment experiences, past meditation experiences, and eating self-monitoring app use. We will also collect data on adverse events, positive and negative affect measured via the Positive and Negative Affect Schedule [55], savoring measured via the Savoring Beliefs Inventory [56], food craving measured via the Food Cravings Questionnaire-Trait-reduced [57], anxiety symptoms measured via the Generalized Anxiety Disorder-7 item assessment [58], body satisfaction measured via the Body Shape Questionnaire [59], binge eating symptoms measured via the Binge Eating Scale [60], and BMI derived from self-reported height and weight.

### Procedure

#### Screening

**Figure 2:**
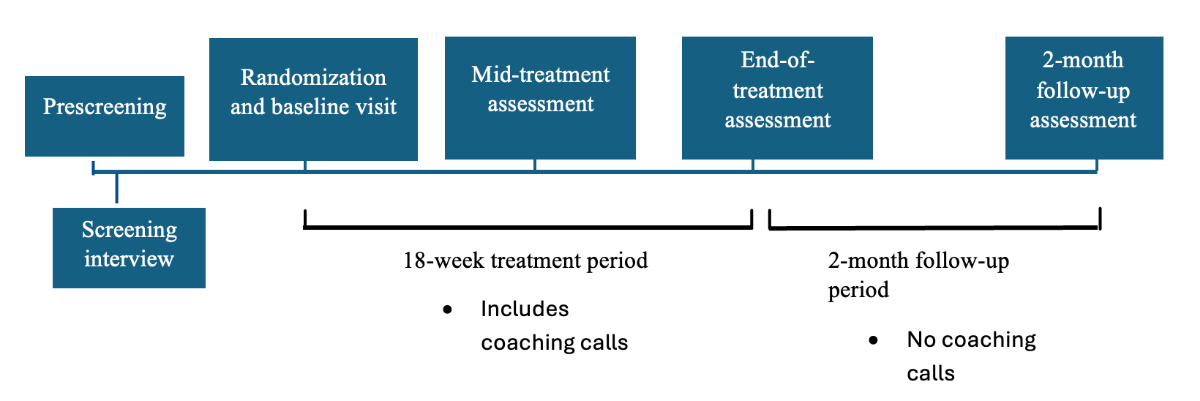
Cognitive behavioral therapy (CBT)–based Mindful Courage timeline. Note: at baseline, participants are randomized to either (1) CBT-based Mindful Courage+coached self-monitoring or (2) coached self-monitoring.

See Figure 2 for a graphic of the study timeline. Online advertisements will include a link for an online prescreening (via HIPAA-secured Qualtrics surveys). Interested participants can then use this link to be assessed for preliminary eligibility; this will include questions on age, ability to engage in the research study, and the Eating Disorder Diagnostic Scale [61]. If individuals meet preliminary eligibility based on the online prescreener, they will be invited to a complete a HIPAA-secured Zoom-based screening interview with a trained psychology student. The Zoom-based screening interview will include informed consent, administration of the Structured Clinical Interview for *Diagnostic and Statistical Manual of Mental Disorders, 5th edition* [49] to confirm BED diagnosis, and a semistructured interview-based assessment to evaluate inclusion and exclusion criteria (refer to the Measures section).

#### Randomization and Baseline Session

If participants are eligible and willing to continue, they will be randomly assigned to either coached self-monitoring or *CBT-based Mindful Courage*+coached self-monitoring by the psychology student who completed the screening interview. Following our prior work [35], randomization will be achieved using a web-based computerized urn randomization program that ensures balance across treatment groups based on sex, age, binge eating severity, and race or ethnicity. Allocation concealment will be ensured because the program reveals assignments only after participant enrollment. After randomization, participants will proceed to the baseline study session (also conducted over Zoom by the psychology student; Multimedia Appendix 2) and will be instructed on how to track their food intake in *Recovery Record.* They will also receive the orientation for phone coaching. Participants randomized to the *CBT-based Mindful Courage*+coached self-monitoring condition will be instructed on how to access *CBT-based Mindful Courage* (either on their computers or via Android or iPhone) and will complete session 1 of the intervention as part of the visit. After completing session 1, participants will be informed that they will have 18 weeks of access to the app to complete 16 lessons and the 2 accompanying mindfulness practices, with weekly support from a student coach.

#### Midtreatment, End-of-Treatment, and 2-Month Follow-Up Assessments

After the baseline visit, participants will complete 3 follow-up assessments: (1) midtreatment assessment (weeks 8-9), (2) end-of-treatment assessment (to be administered within 2 weeks of completing the final lesson or reaching the end of the study period; weeks 16-20), and (3) a 2-month follow-up (2 months after the end of treatment; weeks 24-28). The exact assessment timeline may vary for each participant according to scheduling availability and whether the 16 lessons are completed before the end of the study period.

### Statistical Analysis

#### Feasibility and Acceptability

We will use descriptive statistics to examine feasibility and acceptability of *CBT-based Mindful Courage*. Both groups will be included in analyses of recruitment feasibility, randomization, and assessment completion, whereas only participants in the *CBT-based Mindful Courage* group will be included for evaluating acceptability and feasibility of intervention completion. Specifically, we will compute average scores and frequencies for all feasibility and acceptability benchmarks.

#### Outcomes and Mechanisms

On an exploratory basis, we will evaluate changes in outcomes and mechanisms from baseline to each subsequent assessment point using linear mixed models, separately for participants in each of the conditions (n=20 in each condition). Mixed models enable the inclusion of all participants regardless of missing data (ie, an intent-to-treat analysis). We will also calculate Cohen *d* effect sizes for changes within condition.

## Results

The study was funded in April 2023. Data collection began in November 2025. We anticipate that the recruitment will be completed by November 2026. At the time of initial manuscript submission, no participants had been recruited. We expect results to be analyzed by May 2027 and published by May 2028.

## Discussion

### Anticipated Findings

This protocol describes the design of a pilot RCT with the primary objectives of evaluating the feasibility and acceptability of a digital program combining CBT and mindfulness training for BED—*CBT-based Mindful Courage*. As exploratory objectives, we will evaluate changes in binge eating frequency, global ED symptoms, clinical impairment, and depressive symptoms (as outcomes), as well as emotion dysregulation, dietary overrestriction, and trait mindfulness (as potential mechanisms).

On the basis of our prior research [22,23,32], we anticipate that *CBT-based Mindful Courage* will be acceptable and feasible, setting the stage for an efficacy trial. Our recent study evaluating an 8-week version of *Mindful Courage* for anorexia nervosa and bulimia nervosa concluded that intervention feasibility was excellent and that the intervention was acceptable to participants [23]. Furthermore, our pilot trial evaluating a single-session version of *Mindful Courage* for BED demonstrated excellent acceptability and 100% intervention completion [32]. Preliminary efficacy was demonstrated in both cases, with significant improvements in ED symptoms as well as secondary outcomes (eg, clinical impairment).

### Strengths and Limitations

This study will advance our understanding of the feasibility and acceptability of integrating CBT with mindfulness training for EDs. This trial is also innovative as the first digital intervention for BED to place a major emphasis on mindfulness training. Overall, this study advances our understanding of the viability of digital mindfulness–based treatment as a modality for treating EDs in general, and BED in particular.

This study has several potential limitations. First, a common problem with digital treatments and online research studies is high dropout rates, from both the treatment and study assessments. To overcome this problem, we will implement various retention strategies such as graduated incentives and the inclusion of coaches. Second, we have a relatively low sample size. Nevertheless, the sample size is sufficient to evaluate our primary feasibility and acceptability aims, and this type of test follows the NCCIH model for treatment development [33]. Third, the sample may not be representative of the population.

### Future Directions

Nevertheless, the study has the potential to be an important step in developing a digital treatment that will provide large-scale delivery of a treatment for BED. This project can serve as a foundation for a full-scale efficacy RCT. A digital treatment can offer unprecedented possibilities for dissemination in the real world. Finally, although this study focuses on BED, results from this study could inform the development of a similar intervention for other EDs.

## Appendix

Multimedia Appendix 1 Summary of the 16 core lessons in Cognitive behavioral therapy–based Mindful Courage.

Multimedia Appendix 2 Summary of study measures.

Multimedia Appendix 3 Peer review report from ZAT1 JM (13) - National Center for Complementary and Integrative Health Special Emphasis Panel, NCCIH Training and Education Review Panel (National Institutes of Health, USA).

## Abbreviations

BED: binge eating disorder
CBT: cognitive behavioral therapy
ED: eating disorder
EDE-Q: Eating Disorder Examination Questionnaire
HIPAA: Health Insurance Portability and Accountability Act
MBI: mindfulness-based intervention
NCCIH: National Center for Complementary and Integrative Health
RCT: randomized controlled trial

## Data Availability

The datasets generated or analyzed during this study will be available from the corresponding author on reasonable request
