# Supplementary material for: Combining Digital Cognitive Behavioral Therapy With Mindfulness Training for Binge Eating Disorder: Protocol for a Feasibility Trial
Source: JMIR Res Protoc. 2026 Apr 17;15:e91761. doi: 10.2196/91761 (PMC13135158; doi:10.2196/91761)
Supplement: Multimedia Appendix 1 [file resprot_v15i1e91761_app1.pdf]

Multimedia Appendix 1. Summary of the 16 core lessons in Mindful Courage.

Lesson	Cognitive Behavioral Therapy Content	Mindfulness Content	Mindfulness Practices
1. Getting Started	• Psychoeducation about binge eating disorder (BED), including the role of dietary restraint and difficulties with emotion regulation in BED<br>• Goal-setting<br>• Introduce self-monitoring<br>• Introduce weekly weighting and weight psychoeducation	• Introduce mindfulness<br>• Identify ways in which mindfulness plays a role in BED recovery<br>• Introduce and practice a formal guided mindfulness meditation	Sight, sound, and breath practice (x2)<br><br>Mindfulness practice reflecting on the recovery journey ahead
2. Foundations of Recovery: Regular Eating and Mindfulness	• Trouble-shoot self-monitoring<br>• Explain the link between irregular eating and binge eating<br>• Introduce regular eating<br>• Understand the benefits of regular eating	• Bring awareness to the consequences of irregular eating<br>• Introduce the BOAT (Breath, Observe, Accept, Take a Moment) practice	BOAT practice (x2)<br><br>Sight, sound, and breath practice

	• Learn how to implement and troubleshoot regular eating		
3. Responding with Awareness	• Differentiate between thoughts, emotions, and behaviors<br>• Introduce how thoughts, emotions, and behaviors relate to each other in BED	• Define automatic pilot and its role in eating disorders	BOAT for imagining responding to a difficult situation (x2)<br><br>Sight, sound, and breath practice
4. Mindful Eating	• Trouble-shoot regular eating	• Introduce a brief BOAT practice<br>• Psychoeducation about mindful eating<br>• Introduce a mindful eating practice	Mindful eating practice (x2)<br><br>BOAT practice
5. Hunger and Fullness	• Psychoeducation on creating satiating meals (portion sizes, including a range of macronutrients, etc).<br>• Reducing dietary restraint	• Identify one's hunger cues and avoiding eating when not hungry<br>• Identify one's fullness cues and	Hunger awareness practice (x2)<br><br>Fullness awareness practice

	(food/macronutrient avoidance, dietary rules)	stopping eating when full<br>• Awareness of how eating different foods impacts hunger and fullness	
6. Following Your Values	• Define overvaluation of weight and shape and its consequences<br>• Reduce overvaluation of weight and shape	• Define values and their role in the recovery process<br>• Use mindfulness to clarify one's values<br>• Translate values into action<br>• Explain the ways in which personal values are like a compass that can influence our actions and choices in daily life<br>• Describe ways in which mindfulness and the BOAT can be helpful for making values-based decisions on-the-go	Values awareness practice (x2)<br><br>Brief BOAT practice<br><br>BOAT for values-based decision making
7. Coping with Binge Eating Urges	• Define a binge eating urge<br>• Identify personal triggers (thought,	• Describe the role of mindfulness in coping with binge eating urges	BOAT practice for

	feeling, physical sensation, disordered eating behavior, an event, or an action) that elicit binge eating urges<br>• Learn how to conduct a behavior chain analysis for when binge eating occurs<br>• Learn to identify cues and consequences of binge eating	• Describe ways the BOAT can be helpful for responding skillfully to triggering situations that elicit binge eating urges	food cravings<br><br>Meditation for binge eating urges<br><br>BOAT for binge eating urges (x2)
8. Coping with Difficult Emotions	• Reducing emotional eating by identifying alternative activities and underlying issues that may be driving emotional eating<br>• Problem solving emotional eating	• Practicing mindfulness of one's emotions<br>• Labeling different emotions<br>• Relating to emotions like visitors<br>• Describe the role of difficult emotions in BED<br>• Using mindful awareness and acceptance to cope with difficult	Mindfulness of emotions while reading the Guest House poem<br><br>Mindfulness of emotions<br><br>BOAT for emotions

		emotions without binge eating	
9. Coping with ED Thoughts	• Define ED thoughts<br>• Describe the role of ED thoughts in BED<br>• Practicing breaking food rules	• Recognize unhelpful ED thoughts<br>• Practice mindful awareness and acceptance of ED thoughts<br>• Explain what it means to view thoughts as thoughts and not truths or commands	Walking down the street exercise<br><br>BOAT for ED thoughts<br><br>Mindfulness of thoughts exercise<br><br>Clouds in the sky meditation
10. Practicing Self-Compassion	• Define the role of lapses in BED<br>• Describe the role of self-critical thoughts in the relapse cycle	• Explain how self-compassion can help to cope with lapses and facilitate recovery<br>• Create a brief self-compassion reminder statement<br>• Practice mindfulness while mentally reciting one’s self-compassion reminder	Self-compassion practice<br><br>Self-compassion reminder practice<br><br>BOAT for self-compassion

			Kindness practice

11. Exploring Your Needs	• Define self-care<br>• Describe the role of self-care in recovery	• Describe the type of needs that may underlie one's urge to binge eat<br>• Explain how mindfulness can help us understand our needs and take steps to take care of ourselves without binge eating	Exploring your needs practice<br><br>Brief BOAT practice<br><br>Mindful movement exercise<br><br>Mindful rest exercise
12. Coping with Body Dissatisfaction	• Psychoeducation regarding a healthy body image<br>• Psychoeducation regarding body/shape checking and avoidance<br>• Reducing body/shape checking and avoidance, comparison making<br>• Accepting a normal weight for your body	• Awareness surrounding body checking	BOAT for ED thoughts<br><br>Mindfulness of body image thoughts<br><br>Body scan meditation

13. Being Curious	• Define curiosity	• Practice mindfulness as a way of developing curiosity<br>• Explain the steps of the SOAK (Stop, Observe, Appreciate, Keep Curious).<br>• Describe the role of the SOAK in BED recovery.	SOAK practice (x2)<br><br>SOAK while viewing photos<br><br>SOAK while engaging in a daily activity
14. Savoring Positive Emotions	• Moving reward from food to life<br>• Planning for engaging in specific pleasurable activities other than eating	• Identify different types of pleasant emotions<br>• Use mindfulness to anticipate positive emotions<br>• Use mindfulness to fully engage in positive experiences and appreciate the moment<br>• Use mindfulness to savor positive emotions after they occur<br>• Describe how the BOAT and the SOAK are both	Brief SOAK practice<br><br>Savoring exercise<br><br>SOAK for savoring a positive situation<br><br>Getting to know positive emotions

		mindfulness tools one can use in different situations.	
15. Positive Emotions as Visitors	• Explain how a relentless pursuit of positive emotions can trigger binge eating	• Reflect on the ways in which emotional experiences in life are complex (e.g., pleasant emotions can be mixed together with unpleasant emotions)<br>• Describe the difference between letting positive emotions come and go versus chasing them or clinging to them	Letting positive emotions come and go<br><br>SOAK practice<br><br>SOAK for anticipating a positive situation
16. The Journey Ahead	• Define a lapse vs. relapse<br>• Reflect on factors that may make one vulnerable to a lapse or relapse<br>• Reflect on skills learned in the program that may be helpful when	• Use mindfulness to reflect on the recovery journey<br>• Working to develop a mindfulness practice routine after the treatment is over<br>• Find resources to stay connected to	Reflecting on your recovery journey

	experiencing a lapse/relapse<br>• Have realistic expectations for recovery	mindfulness practice in the future	
