# Supplementary material for: Combining Digital Cognitive Behavioral Therapy With Mindfulness Training for Binge Eating Disorder: Protocol for a Feasibility Trial
Source: JMIR Res Protoc. 2026 Apr 17;15:e91761. doi: 10.2196/91761 (PMC13135158; doi:10.2196/91761)
Supplement: Multimedia Appendix 2 [file resprot_v15i1e91761_app2.pdf]

Multimedia Appendix 2. Summary of study measures

Construct	Measurement Instrument	Interview (I) or Self-Report (S/R)	Pre-Screening	Screening Interview	Randomization and Baseline Visit	Mid-Treatment Assessment	End-of-Treatment Assessment	2-Month Follow-Up Assessment
**Eating Disorders**	Eating Disorder Diagnostic Scale [1]	S/R	x					
**Psychiatric Diagnosis**	Structured Clinical Interview for DSM-V [2]	I		x				
**Anxiety Symptoms**	Generalized Anxiety Disorder-7 [3]	S/R			x	x	x	x
**Frequency of Objective Binge Eating Episodes**	Eating Disorder Examination-Questionnaire [4]	S/R			x	x	x	x
**Clinical Impairment**	Clinical Impairment Assessment [5]	S/R			x	x	x	x
**Depressive Symptoms**	Patient Health Questionnaire-9 [6]	S/R			x	x	x	x

**Trait Mindfulness**	Cognitive and Affective Mindfulness Scale-Revised [7]	S/R			x	x	x	x
**Emotion Regulation**	Difficulties in Emotion Regulation Scale-Short Form [8]	S/R			x	x	x	x
**Dietary Over-Restriction**	Eating Disorder Examination-Questionnaire Restraint Subscale [4]	S/R			x	x	x	x
**Positive and Negative Affect**	Positive and Negative Affect Schedule [9]	S/R			x	x	x	x
**Savoring**	Savoring Beliefs Inventory [10]	S/R			x	x	x	x
**Food Craving**	Food Cravings Questionnaire-Trait-reduced [11]	S/R			x	x	x	x
**Binge Eating Symptoms**	Binge Eating Scale [12]	S/R			x	x	x	x
**Body Satisfaction**	Body Shape Questionnaire [13]				x	x	x	x

**Acceptability**	System Usability Scale [14]	S/R					x[a]	

[a]*CBT-based Mindful Courage*+coached self-monitoring only

References

1. Stice E, Telch CF, Rizvi SL. Development and validation of the Eating Disorder Diagnostic Scale: a brief self-report measure of anorexia, bulimia, and binge-eating disorder. *Psychol Assess*. 2000;12(2):123-131. doi:10.1037//1040-3590.12.2.123
2. First MB, Williams JBW, Karg RS, Spitzer RL. *Structured Clinical Interview for DSM-5 Disorders: Research Version (SCID-5-RV)*. American Psychiatric Association; 2015.
3. Spitzer RL, Kroenke K, Williams JBW, Löwe B. A Brief Measure for Assessing Generalized Anxiety Disorder: The GAD-7. *Archives of Internal Medicine*. 2006;166(10):1092-1097. doi:10.1001/archinte.166.10.1092
4. Fairburn CG, Beglin SJ. Eating Disorder Examination Questionnaire (EDE-Q 6.0). In: Fairburn CG, ed. *Cognitive Behavior Therapy and Eating Disorders*. Guilford Press; 2008:309-313.
5. Bohn K, Doll HA, Cooper Z, O'Connor M, Palmer RL, Fairburn CG. The measurement of impairment due to eating disorder psychopathology. *Behaviour Research and Therapy*. 2008;46(10):1105-1110. doi:10.1016/j.brat.2008.06.012
6. Kroenke K, Spitzer RL, Williams JBW. The PHQ-9. *Journal of General Internal Medicine*. 2001;16(9):606-613. doi:10.1046/j.1525-1497.2001.016009606.x
7. Feldman G, Hayes A, Kumar S, Greeson J, Laurenceau JP. Mindfulness and Emotion Regulation: The Development and Initial Validation of the Cognitive and Affective Mindfulness Scale-Revised (CAMS-R). *J Psychopathol Behav Assess*. 2007;29(3):177-190. doi:10.1007/s10862-006-9035-8
8. Kaufman EA, Xia M, Fosco G, Yaptangco M, Skidmore CR, Crowell SE. The Difficulties in Emotion Regulation Scale Short Form (DERS-SF): Validation and Replication in Adolescent and Adult Samples. *J Psychopathol Behav Assess*. 2016;38(3):443-455. doi:10.1007/s10862-015-9529-3
9. Watson D, Clark LA, Tellegen A. Development and validation of brief measures of positive and negative affect: The PANAS scales. *Journal of Personality and Social Psychology*. 1988;54(6):1063-1070. doi:10.1037/0022-3514.54.6.1063
10. Bryant F. Savoring Beliefs Inventory (SBI): A scale for measuring beliefs about savouring. *Journal of Mental Health*. 2003;12(2):175-196. doi:10.1080/0963823031000103489
11. Cepeda-Benito A, Gleaves DH, Williams TL, Erath SA. The development and validation of the state and trait food-cravings questionnaires. *Behavior Therapy*. 2000;31(1):151-173. doi:10.1016/S0005-7894(00)80009-X
12. Gormally J, Black S, Daston S, Rardin D. The assessment of binge eating severity among obese persons. *Addictive Behaviors*. 1982;7(1):47-55. doi:10.1016/0306-4603(82)90024-7
13. Cooper PJ, Taylor MJ, Cooper Z, Fairbum CG. The development and validation of the body shape questionnaire. *International Journal of Eating Disorders*. 1987;6(4):485-494. doi:10.1002/1098-108X(198707)6:4%3C485::AID-EAT2260060405%3E3.0.CO;2-O
14. Brooke J. SUS: A "Quick and Dirty" Usability Scale. In: Jordan PW, Thomas B, Weerdmeester A, McClelland AL, eds. *Usability Evaluation In Industry*. Taylor and Francis; 1996.
