# Supplementary material for: Combining Digital Cognitive Behavioral Therapy With Mindfulness Training for Binge Eating Disorder: Protocol for a Feasibility Trial
Source: JMIR Res Protoc. 2026 Apr 17;15:e91761. doi: 10.2196/91761 (PMC13135158; doi:10.2196/91761)
Supplement: Multimedia Appendix 3 [file resprot_v15i1e91761_app3.pdf]

SUMMARY STATEMENT
(Privileged Communication)

PROGRAM CONTACT:
Dr. Lanay Mudd
301-594-9346
lanay.mudd@nih.gov

Release Date: 12/01/2022
Revised Date:

Application Number: 1 K23 AT012126-01A1
Formerly: 1K23AT012126-01

Principal Investigator

SALA, MARGARITA

Applicant Organization: YESHIVA UNIVERSITY

Review Group: ZAT1 JM (13)
National Center for Complementary and Integrative Health Special Emphasis Panel
NCCIH Training and Education Review Panel (CT)

Meeting Date: 11/09/2022
Council: JAN 2023
Requested Start: 04/01/2023
RFA/PA: PA20-206
PCC: MUDDL
Dual PCC: 8K-RTTTA
Dual IC(s): MH

Project Title: Digital Mindfulness Meditation-enhanced Cognitive Behavioral Therapy (CBT-MM) for Binge Eating Disorder
SRG Action: Impact Score:20
Next Steps: Visit https://grants.nih.gov/grants/next_steps.htm
Human Subjects: 30-Human subjects involved - Certified, no SRG concerns
Animal Subjects: 10-No live vertebrate animals involved for competing appl.
Gender: 1A-Both genders, scientifically acceptable
Minority: 1A-Minorities and non-minorities, scientifically acceptable
Age: 7A-Only Adults, scientifically acceptable

Project Year	Direct Costs Requested	Estimated Total Cost
1	147,329	159,115
2	150,785	162,848
3	154,668	167,041
4	158,162	170,815
5	158,027	170,669
TOTAL	768,971	830,488

ADMINISTRATIVE BUDGET NOTE: The budget shown is the requested budget and has not been adjusted to reflect any recommendations made by reviewers. If an award is planned, the costs will be calculated by Institute grants management staff based on the recommendations outlined below in the COMMITTEE BUDGET RECOMMENDATIONS section.

1K23AT012126-01A1 Sala, Margarita

RESUME AND SUMMARY OF DISCUSSION: This K23 resubmission application titled "Digital Mindfulness Meditation-enhanced Cognitive Behavioral Therapy (CBT-MM) for Binge Eating Disorder" is submitted in response to PA-20-206 "Mentored Patient-Oriented Research Career Development Award (Parent K23 Independent Clinical Trial Required) by Yeshiva University with Dr. Margarita Sala as the candidate. This application proposes to develop, refine, and test the feasibility and acceptability of and within-group changes following a novel digitally delivered, combined mindfulness meditation (MM) and cognitive behavioral therapy (CBT) intervention for binge eating disorder (BED). The candidate is an assistant professor and currently leads the Mindfulness, Eating Disorders, and Acceptance Laboratory (MEAL) which focuses on the development of mindfulness-based treatments for eating and weight disorders (EWDs), which builds upon her prior related training and well prepares her for the proposed research. Thus, she has a strong commitment toward this area of research which is coupled with a strong record of productivity demonstrated by her ability to secure funding in the past and nearly 40 peer-reviewed publications, over half of which are as first author. Moreover, strong reference letters attest to the candidate's dedication to and potential for development as an independent researcher. The mentorship team includes experienced mentors with relevant expertise in EWDs, intervention development, mindfulness, digital interventions, qualitative research, clinical trials, and statistical analysis. Notably, the team is now more complementary and includes a balance of mentors with whom the candidate has previously worked as well as new mentors that could enable new collaborations. The environment provides the necessary facilities and resources to conduct the proposed research while there is clear assurance from the institution that the candidate will have protected time to complete the proposed research and career development activities. Overall, the career development plan focuses on training in four main domains including developing and refining mindfulness-based treatments for eating disorders, digital interventions, clinical trials, and qualitative methods. Development in these areas would be accomplished with an appropriate combination of workshops, courses, conference attendance, and mentored training. Not only is the scope appropriate, but there is a plan in place for tracking the candidate's progress toward reaching her career development goals and achieving independence. However, there is a question as to the extent to which the candidate needs additional development in mindfulness-based treatments and clinical trials given her strong foundation in these areas. On the other hand, whether the plan will provide sufficient training in digital interventions and if the candidate would also benefit from training in implementation science to support the integration of the intervention into primary care workflows are also of question. Overall, the well-designed research plan proposes an innovative, scalable approach to address an important gap in treatments for BED by targeting both dietary over-restriction and emotion dysregulation through the integration of two evidence-based strategies, MM and CBT, into a digitally delivered intervention. Moreover, the rationale for the proposed study is built upon strong preliminary data from the candidate showing the effect of a brief digitally delivered MM intervention for eating disorders on emotional dysregulation and the content of the intervention is based on other manualized MM-based therapies. Based on prior work, there are also clear benchmarks for feasibility and acceptability. The proposed research is well aligned with the candidate's overall career objectives and could serve as a vehicle for developing an independent program of research focused on developing and evaluating mindfulness-based treatments for EWDs. While modifications to the proposed research plan are responsive to the prior review, some minor concerns did remain. For example, there was a concern regarding the potential appropriateness of the digital CBT control intervention given that the CBT component of the combined CBT-MM appears to be different. How exactly stakeholders would be engaged during the development of the intervention could have been more clearly articulated. More detail could have been given regarding how individuals providing the coaching component of the intervention would be trained or monitored. Finally, there are some questions regarding the ability of the team to recruit nationally and the proposed length of recruitment. In conclusion, this K23 resubmission application is from an exceptional candidate with outstanding support from her institution and mentorship team with a strong career development plan and research plan to support her career progression toward independence.

Overall, there is strong enthusiasm for this application particularly given the responsiveness to the prior review resulting in a more well-rounded mentorship team, clear institutional support, better aligned career development and research plans, and improvements in the research plan itself.

DESCRIPTION (provided by applicant): Binge eating disorder (BED) is a prevalent disorder, affecting 3.5% of Americans. BED is maintained by two- pathways: (1) dietary over-restriction and (2) emotion dysregulation. Current treatments for BED, such as Cognitive Behavioral Therapy (CBT) produce suboptimal results because they only target over-restriction. Mindfulness meditation (MM) targets emotion dysregulation and could thus be an important addition to CBT for Eating Disorders. Furthermore, digital delivery of an MMT for BED would offer unprecedented scalability, but there are no current digital MMTs for BED. The current K23 proposal involves developing a digital Mindfulness Meditation-enhanced Cognitive Behavioral Therapy (CBT-MM) for BED via two phases. CBT-MM will target the two different mechanisms of BED (dietary over-restriction and emotion dysregulation). In Phase 1, I will finalize the development of digital CBT-MM via interview-based user testing. In Phase 2, I will conduct a pilot feasibility randomized controlled trial (RCT). For the RCT, 40 individuals with BED recruited nationally will be randomized to digital: 1) CBT-MM (n = 20); or 2) an already developed digital CBT treatment (iTakeControl) only (n = 20). The RCT will be conducted virtually, with video-based assessments at baseline, mid- and post-treatment, and 3-month follow up. Aim 1 is to develop digital CBT-MM. Aim 2 is to evaluate feasibility and acceptability. Aim 3 is to measure within-group changes from pre-to post treatment on binge eating remission, binge eating frequency, weight, quality of life, depressive symptoms, emotion dysregulation, dietary over-restriction, and trait mindfulness. This project has the potential for developing an innovative, theory-driven, efficacious, and scalable mindfulness-based treatment for BED that can have a large public health impact. To carry out this K23, I will receive mentorship from a leading team of experts in the areas of mindfulness, CBT for eating disorders, technology-based treatment development, and qualitative methods. This K23 will specifically facilitate my training in: (1) developing and refining mindfulness-based eating and weight disorder treatments; (2) technology-based treatment development, (3) formalized training in conducting clinical trials, and (4) qualitative methods for intervention development. This award will serve as a launching point for my career as I leverage the training and research skills afforded by this award to become a leader in the field of digital mindfulness-based treatment development for eating and weight disorders.

PUBLIC HEALTH RELEVANCE: I propose to develop a novel digital treatment, Mindfulness Meditation-enhanced Cognitive Behavioral Therapy (CBT-MM), that integrates MM and CBT for the treatment of Binge Eating Disorder (BED) to target the two important mechanisms in the development and maintenance of BED: dietary over-restriction and emotion dysregulation. The current K23 proposal involves developing digital CBT-MM for BED and conducting a pilot randomized control trial to evaluate its feasibility, acceptability, and within-group changes from pre-to post treatment on indices of: binge eating remission, binge eating frequency, weight, quality of life, depressive symptoms, emotion dysregulation, dietary over-restriction, and trait mindfulness. This innovative research project has the potential to advance research on mindfulness-based treatment for eating disorders.

CRITIQUE 1

Candidate: 1
Career Development Plan/Career Goals /Plan to Provide Mentoring: 3
Research Plan: 3
Mentor(s), Co-Mentor(s), Consultant(s), Collaborator(s): 2
Environment Commitment to the Candidate: 1

Overall Impact: This is a K23 resubmission application from Dr. Margarita Sala who is currently an assistant professor at the School of Psychology at Yeshiva University. The candidate proposes to develop a digital intervention that integrates mindfulness meditation (MM) and cognitive behavioral

therapy (CBT) for treating binge eating disorder (BED). The goal is to build an intervention that targets two key mechanisms in the development and maintenance of BED: dietary over-restriction and emotion dysregulation. Aim 1 is to conduct three waves of interview-based user testing (n=5 per wave; total n=15) with 18-65 year-old participants who meet the Diagnostic and Statistical Manual of Mental Disorders, Fifth Edition (DSM-5) criteria for BED; Aim 2 is to conduct a pilot feasibility randomized controlled trial (RCT) with 40 participants with BED who will be randomized to either the digital integrated intervention or to a control digital CBT (treatment duration: 10 weeks). Aim 3 is to assess within-group changes from pre- to post-treatment on BED remission, binge eating frequency, emotion dysregulation, dietary over-restriction, and other mechanisms of change. Key strengths include: 1) a highly productive candidate with a promising future in the field of mindfulness-based interventions (MBIs) for eating disorders; 2) an excellent training team with a balance of mentors with whom the candidate has worked before and mentors with whom the candidate can establish new collaborative relationships; 3) strong institutional support with substantial resources that are not contingent on funding for the proposed project; and 4) a research project that has the potential to close an important gap in treatments for BED by developing a novel intervention that will leverage technology to integrate two evidence-based approaches to treatment: CBT and MM. Weaknesses include: 1) the intervention to be developed includes several components with no plans to systematically optimize the selection and intensity of these components; 2) the career development plan does not include sufficient training in digital interventions; and 3) the candidate would benefit from training in implementation science to support the integration of the new digital intervention in primary care workflows. Still, the strengths outweigh the weaknesses of the proposed career development activities and research project, which have potential for high impact.

1. Candidate:
Strengths
- The candidate has experience leading a research laboratory: Dr. Sala serves as the director of the Mindfulness, Eating Disorders, and Acceptance Laboratory at Yeshiva University; this laboratory focuses on developing mindfulness-based treatments for individuals with eating and weight disorders (EWDs).
- The candidate has experience with mentorship: Dr. Sala is currently supervising seven graduate students, three undergraduate students, and two part-time research assistants.
- The candidate has both clinical and research expertise related to the role of mindfulness in EWDs: Dr. Sala has over seven years of experience conducting research with EWDs and over six years of experience conducting mindfulness research. In addition, Dr. Sala has treated over 50 individuals with EWDs with MBI protocols.
- Dr. Sala received post-doctoral training at the Center for Weight, Eating, and Lifestyle Sciences (WELL Center) at Drexel University, with Dr. Evan Forman (primary mentor on the proposed K23 award application) as her primary mentor.
- The candidate is highly productive: Dr. Sala graduated in 2020 and she already has close to 40 peer-reviewed publications, over half as first author.
- Dr. Sala has experience conducting small-scale clinical trials which will help in achieving the proposed program objectives.

Weaknesses
- No major weaknesses noted.

2. Career Development Plan/Career Goals & Objectives:
Strengths
- Dr. Sala received extensive training in MBIs and eating disorders which will serve as a strong foundation for the proposed work.
- The proposed career development program includes a combination of formal training, conferences, and mentorship.

Weaknesses

- Training in the development of digital interventions is critical for the proposed research project, but the proposed formal training in this domain is relatively narrow, including only one user-centered design workshop.
- The candidate would benefit from training in implementation science to support the integration of the new digital intervention in primary care workflows.
- The career development program focuses on four domains: 1) mindfulness-based treatments for eating disorders; 2) digital interventions; 3) clinical trials; and 4) qualitative methods. Given Dr. Sala's expertise in mindfulness-based treatments for eating disorders and experience with conducting small-scale clinical trials, training in domains 1) and 3) may not be necessary.

3. Research Plan:
Strengths
- Existing treatments for BED produce suboptimal results, partly because they target only one key mechanism, over-restriction, via CBT. This project represents an important step in closing this gap by developing a novel digital treatment that integrates MM with CBT for treating BED.
- The digital intervention will include 1) behavioral elements from CBT for EDs that have been shown to be the most efficacious for targeting dietary over-restriction (e.g., regular meals, self-monitoring) and 2) MM elements to target emotion dysregulation.
- Digital delivery of the integrated intervention can increase treatment access and scalability.
- The control digital CBT (iTakeControl) which will be used in the current project was already developed by Dr. Forman (primary mentor).
- The recruitment/enrollment phase of the pilot RCT (N=40) will be 24 months, enrolling ~2 participants per month, which seems feasible.
- Participants in the control (CBT) arm will be asked about any MM practice they may have engaged in to assess for treatment contamination.
- Benchmarks for feasibility and acceptability are specified based on prior evidence relating to MM treatment and digital CBT for ED.

Weaknesses
- The intervention includes multiple components: 1) weekly CBT modules; 2) weekly MM modules; 3) daily 30-minute MM; 4) weekly coaching calls (10-15 minutes) to promote engagement and provide technical support; 5) three-four text reminders per week to engage in the app; 6) additional phone/text contact for participants not engaging in the app. The long-term plan (in an R01 award application) is to confirm the effectiveness of this intervention package relative to control; but there is no plan to systematically optimize the selection and intensity of the components.
- Dr. Sala considered a three-arm trial comparing treatment as usual versus digital CBT versus digital CBT-MM. However, she deemed this three-arm not to be necessary because there are already data on the control digital CBT. If there are already data on the control digital CBT, then there is no clear justification for including it in the proposed trial and resources should be allocated to testing the feasibility/acceptability of other controls such as digital MM alone.
- It's not clear how the new intervention can be integrated into existing primary care workflows.

4. Mentor(s), Co-Mentor(s), Consultant(s), Collaborator(s):
Strengths
- Dr. Forman (Drexel University; primary mentor) has experience in designing, refining, implementing, and evaluating innovative behavioral treatments (including acceptance-based treatments) for obesity and related eating problems. Dr. Forman also has extensive expertise in conducting clinical trials, including virtual clinical trials.
- Dr. Forman served as primary or co-mentor on several National Institutes of Health (NIH)-funded training awards.
- Dr. Feldman (Yeshiva University; co-mentor) has extensive training in intervention development and conducting clinical trials.

- Dr. Kober (Yale; co-mentor) has worked on several projects that tested the feasibility, acceptability, and efficacy of novel MBIs, and investigated their psychological and neural mechanisms. Dr. Kober also has experience in digital MBIs.
- Dr. Aslan (Yale; co-mentor) brings expertise in analyzing data from clinical trials.
- Dr. Bricker (Fred Hutchinson Cancer Research Center; co-mentor) brings expertise in digital interventions.
- Dr. Hay (Sloan Kettering Cancer Center; co-mentor) brings expertise in qualitative methodologies.
- The mentorship team is a balance of mentors with whom Dr. Sala has worked before (Drs. Forman, Feldman, Kober, and Aslan) and mentors with whom Dr. Sala can establish collaborative relationships in the future (Drs. Bricker and Hay).

Weaknesses

- There is overlap in expertise: both Dra. Forman and Kober bring expertise in MBIs (in-person and digital).
- Dr. Feldman's research focuses primarily on reducing disparities in asthma outcomes, hence their experience in large-scale randomized trials may be less relevant to the current project which focuses on eating disorders.

5. Environment:

Strengths

- The Ferkauf Graduate School of Psychology at Yeshiva University, where Dr. Sala is currently an assistant professor (tenure-track; starting 2021), provides an excellent institutional environment for this proposed K23 award.
- Dr. Sala has a laboratory within the school, The Mindfulness, Eating, and Acceptance Laboratory (MEAL), where she mentors several graduate students, has several undergraduate research assistants volunteering, and two part-time research assistants employed.
- The letter from the Dean of the Graduate School of Psychology at Yeshiva University is very strong, emphasizing that institutional commitment to Dr. Sala is not contingent upon the receipt of this proposed K23 award.
- The standard teaching load in psychology is five courses per year. The letter from the Dean clarifies that if the K23 is awarded, Dr. Sala's teaching load will be reduced to one course per year. In addition, Dr. Sala does not have direct patient care or clinical supervision responsibilities and hence will be able to dedicate time to training and research.

Weaknesses

- No major weaknesses noted.

Study Timeline:

Strengths

- Study timeline is adequate

Weaknesses

- No major weaknesses noted.

Protections for Human Subjects

Acceptable Risks and Adequate Protections

Data and Safety Monitoring Plan (Applicable for Clinical Trials Only):

- Acceptable

Inclusion Plans

- Sex/Gender: Distribution justified scientifically
- Race/Ethnicity: Distribution justified scientifically
- For NIH-Defined Phase III trials, Plans for valid design and analysis: Not applicable
- Inclusion/Exclusion Based on Age: Distribution justified scientifically

Vertebrate Animals
Not Applicable (No Vertebrate Animals)

Biohazards
Not Applicable (No Biohazards)

Resubmission
- Responses to prior review comments are adequate.

Training in the Responsible Conduct of Research
Acceptable
Comments on Format:
- The format is acceptable.

Comments on Subject Matter:
- The subject matter is acceptable.

Comments on Faculty Participation:
- Faculty participation is acceptable.

Comments on Duration:
- The duration is acceptable.

Comments on Frequency:
- The frequency is acceptable.

Select Agents
Not Applicable (No Select Agents)

Resource Sharing Plans
Acceptable

Authentication of Key Biological and/or Chemical Resources
Not Applicable (No Relevant Resources)

Budget and Period of Support
Recommend as Requested

CRITIQUE 2

Candidate: 1
Career Development Plan/Career Goals /Plan to Provide Mentoring: 1
Research Plan: 3
Mentor(s), Co-Mentor(s), Consultant(s), Collaborator(s): 1
Environment Commitment to the Candidate: 1

Overall Impact: This is a K23 resubmission application by a highly successful and productive candidate who aims to further her training and research experience in developing and evaluating mindfulness-based treatments for eating and weight disorders (EWD). For the research plan, Dr. Sala proposes to develop a digital mindfulness meditation-enhanced cognitive behavioral therapy (CBT-MM) intervention and evaluate its feasibility and acceptability in a small pilot randomized controlled trial (RCT) (n=40). Exploratory analyses will assess within-group changes in binge eating disorder (BED)-related outcomes compared with a control digital CBT. She provides a strong rationale for this approach; namely that interventions combining CBT and MM have not been tested for BEDs previously,

although MM approaches have shown promise in improving emotional dysregulation, an important pathway through which BEDs are maintained. The proposed intervention will build from the candidate's pilot work developing a brief digital MM intervention for ED, and will undergo refinement, initially with patient and provider stakeholders, and subsequently through iterative evaluations with study participants. The second phase will test the refined CBT-MM intervention in a small pilot RCT (n=40). Proposed training and career development activities are clearly outlined, include a combination of structured (e.g., workshops, courses) and unstructured (e.g., independent work with mentors) experiences, and align with the candidate's short- and long-term research goals, positioning her well for success as an independent investigator. She has glowing recommendations from references, and extremely strong letters of support from her mentoring team. Institutional support is also strong. This resubmission was very responsive to the prior review and includes modifications to the mentoring team, career development goals and activities, study design, and study outcomes, among other changes. The proposed research is rigorous and innovative and addresses an important gap regarding effective treatment approaches for BED. The career development plan is comprehensive and feasible and fills gaps in the candidate's research and training experiences. Completion of this work will position the candidate well for success as an independent investigator. Weaknesses concern the CBT approach used in the intervention versus the control condition, the lack of clarity regarding the involvement of stakeholders in the development of the intervention, and the lengthy recruitment time. In sum, this is an outstanding application from a very strong candidate who shows great promise for making important contributions to the field.

1. Candidate:
Strengths
- Dr. Sala has demonstrated excellent productivity for this stage in her career. She has 39 peer-reviewed publications, including over half as first author, and received considerable funding during her graduate studies (e.g., National Science Federation Graduate Research Fellowship; >$150K from various sources).
- Dr. Sala's prior training in mindfulness-based treatments and EWDs has prepared her well for the proposed research, and she appears to have strong quantitative skills (quantitative minor).
- Dr. Sala's mentor letters are exceedingly strong and convey enthusiasm for the candidate and confidence in her ability to develop into an independent investigator.
- Dr. Sala's reference letters are absolutely glowing, with two saying that she was the best student they have ever worked with. They all highlight her passion, drive, ability to work efficiently and independently and with little guidance, and productivity.

Weaknesses
- No major weaknesses noted.

2. Career Development Plan/Career Goals & Objectives:
Strengths
- Dr. Sala has identified four key areas of growth addressed by the career development and research activities to move her towards an independent research program in developing and evaluating digital mindfulness-based therapies (MBTs) for EWDs: 1) developing and refining MBTs for EWDs, 2) technology-based treatment development, 3) formalized training in conducting clinical trials, and 4) qualitative methods for intervention development.
- These areas help to address gaps in Dr. Sala's experience and training and will position her well to achieve scientific independence.
- Dr. Sala's career development plan includes a combination of workshops and courses, conferences, independent work with mentors, and participation in ongoing mentor activities. These are appropriate in scope and content for achieving her research and career development goals.
- Primary mentor, Dr. Forman, outlines plans for quarterly and annual evaluations of Dr. Sala's progress.

Weaknesses
- No major weaknesses noted.

3. Research Plan:
Strengths
- The proposed research is innovative in its focus on combining two treatment approaches, CBT and MM, that have not been previously tested in RCTs together, for improving treatment outcomes for BED.
- The mixed methods approach will make an important contribution toward the refinement of the MM-CBT intervention.
- The digital delivery format for this type of intervention is also innovative and stands to improve reach and scalability.
- Preliminary pilot data conducted by the candidate shows the effects of a brief digital mindfulness meditation-based therapy (MMT) for ED on emotional dysregulation.
- The content of the proposed CBT-MM intervention builds upon other manualized MMT interventions.
- There are very clear feasibility and acceptability benchmarks based on prior MMT and digital CBT interventions for ED.
- There is thoughtful consideration of alternative design issues and a strong rationale for the procedures proposed in the application.

Weaknesses
- It is unclear how similar/different the CBT component will be across the two interventions. The digital CBT intervention (control) appears to use an established app-based CBT intervention developed by one of the mentors (Dr. Forman), whereas the CBT component for the digital CBT-MM intervention appears to draw from multiple CBT interventions. This could have implications for participants' responses and outcome if the CBT components are different.
- Although a brief mention of involving patients and providers in Phase 1 is added in this resubmission, it is unclear how they will be engaged and contribute to the development of the MMT intervention.
- Concern remains regarding the progress of recruitment (Phase 1: 15 participants in nine months; Phase 2: 40 participants in two years), and it's not clear that the candidate or mentors have experience with the proposed national recruitment strategies.

4. Mentor(s), Co-Mentor(s), Consultant(s), Collaborator(s):
Strengths
- The primary mentor, Dr. Forman, has strong mentoring experience including serving as primary or co-mentor on multiple National Institutes of Health (NIH)-funded training awards (F and K) and is currently serving as a mentor for three NIH-funded training grants. He also has extensive expertise in mindfulness-based interventions for EWDs.
- The mentoring team is comprised of faculty with complementary expertise in the areas outlined for Dr. Sala's research and career developments needs including mindfulness-based treatment development (Dr. Kober), technology-based treatment development (Dr. Bricker), conduct of clinical trials (Dr. Feldman), qualitative methods (Dr. Hay), and biostatistics (Dr. Aslan).
- There are clear plans for regular meetings with the mentors.
- The primary mentor, Dr. Forman, outlines plans to evaluate Dr. Sala's progress on a quarterly basis with more formal annual evaluations.

Weaknesses
- No major weaknesses noted.

5. Environment:
Strengths

- A strong letter of institutional support is provided by the Dean of Psychology at Yeshiva University. Importantly, Dr. Sala's teaching load will be one course per year, compared with the typical five courses per year.
- The Dean's letter indicates appropriate administrative support and research space and equipment available to Dr. Sala.

Weaknesses
- No major weaknesses noted.

Study Timeline:
Strengths
- The study milestones are clearly delineated.

Weaknesses
- The proposed time devoted to recruitment seems unnecessarily long.

Protections for Human Subjects
Acceptable Risks and Adequate Protections
- This is a low-risk study with adequate protection against risks.

Data and Safety Monitoring Plan (Applicable for Clinical Trials Only):
- Acceptable
  - This study will form an Independent Safety Monitoring Committee to monitor participant safety and assess study progress.

Inclusion Plans
- Sex/Gender: Distribution justified scientifically
- Race/Ethnicity: Distribution justified scientifically
- For NIH-Defined Phase III trials, Plans for valid design and analysis: Not applicable
- Inclusion/Exclusion Based on Age: Distribution justified scientifically
- This study proposes to recruit 75% White, 18% Black, and 24% Hispanic men and women (40% men).

Vertebrate Animals
Not Applicable (No Vertebrate Animals)

Biohazards
Not Applicable (No Biohazards)

Resubmission
- This resubmission application is extremely responsive to previous reviewers' comments. The candidate removed/added mentors to add diversity to her mentorship team; modified training goals and activities to more closely align with her proposed research activities and long-term goals; changed the intervention delivery to a digital format and expanded on the rationale to elaborate on significance and enhance innovation; modified the primary outcome to focus on feasibility; and made additional changes.

Training in the Responsible Conduct of Research
Acceptable
Comments on Format:
- The candidate proposes responsible conduct of research (RCR) training through in-person and online courses and through individual discussions with her mentors.

Comments on Subject Matter:
- All relevant subject matter is included.

Comments on Faculty Participation:

- Faculty involvement primarily through individual discussions is outlined.

Comments on Duration:

- Training in RCR will take place throughout the full five years of the proposed award period.

Comments on Frequency:

- The frequency of training varies by format and is reasonable.

Resource Sharing Plans
Acceptable

- The Data Sharing Plan describes adhering to NIH policies.

Budget and Period of Support
Recommend as Requested

CRITIQUE 3

Candidate: 1
Career Development Plan/Career Goals /Plan to Provide Mentoring: 1
Research Plan: 3
Mentor(s), Co-Mentor(s), Consultant(s), Collaborator(s): 1
Environment Commitment to the Candidate: 2

Overall Impact: Dr. Sala is a well-trained and productive researcher who aims to focus her career on developing and evaluating mindfulness-based treatments for eating and weight disorders (EWDs). She has assembled an excellent mentoring team and has strong institutional support. The proposed project works towards evaluating the hypothesis that mindfulness meditation (MM) could be an important therapeutic complement to cognitive behavioral therapy (CBT) in the treatment of binge eating disorders (BEDs), by addressing emotional dysregulation (in addition to CBT's impact on dietary over-restriction). The proposed research incudes 1) finalizing the development of a novel digital intervention combining CBT and MM for BED, 2) evaluating feasibility and acceptability, and measuring within-group changes using a variety of relevant validated measures. The career development plan includes specific training related to topics of importance for Dr. Sala's career goals (e.g., developing mind body treatment for EWDs, development of a technology-based intervention, and training conducting clinical trials and qualitative research). The research plan is well-developed, includes appropriate eligibility and outcome criteria, as well as appropriate plans for intervention delivery and outcome assessment. It could benefit from additional details about how the candidate's prior recruitment experiences dovetail with the proposed plan and how recruitment could be augmented if initial plans prove ineffective. Likewise, additional information on the coaching component to the intervention would be helpful.

1. Candidate:
Strengths

- Dr. Sala is a well-trained and productive researcher with a PhD degree in clinical psychology, from Southern Methodist University, a clinical internship at the West Haven Department of Veterans Affairs (VA) in Connecticut, and the completion of a postdoctoral fellowship at Drexel University.
- She has secured prior research funding such as a National Science Federation Graduate Research Fellowship, won research awards such as the American Psychological Association Dissertation Research Award, and has 39 publications with an H-index of 16.
- Both her written application and letters of support suggest a deep commitment to a career in research and a high likelihood of becoming an independent investigator.
- The proposed work is a logical extension of her prior efforts, suggesting that she is well prepared to organize and manage it.

- A plan is outlined for training in data management (Design and Conduct of Clinical Research course at the Albert Einstein College of Medicine as well as mentoring by Dr. Aslan and Dr. Hays).

Weaknesses

- The candidate has minimal prior training in data management, but there is an appropriate plan to remedy that concern.

2. Career Development Plan/Career Goals & Objectives:

Strengths

- The career development plan identifies distinct and relevant areas for training that seem likely to contribute to the candidate's scientific development towards being an independent investigator (e.g., developing mind-body treatments for EWDs, development of a technology-based intervention, and training conducting clinical trials and qualitative research).
- The plan includes an appropriate mix of formal training (e.g., Mind and Life Summer Research Institute on mindfulness, a National Institutes of Health (NIH) Summer Institute on Randomized Behavioral Clinical Trials, a Brown Qualitative Science and Methods Program, workshops (e.g., user-centered design), and mentoring with regular meetings with committed mentors. Both qualitative and quantitative methods are incorporated.
- The primary mentor will provide informal quarterly evaluations and more formal annual evaluations of progress.

Weaknesses

- The process for evaluating progress is vague. However, given her primary mentors established track record and assurance of evaluations, this is considered a minor point.

3. Research Plan:

Strengths

- The candidate provides a strong justification for the rationale and innovation of her approach, and the relevant gap in the literature suggesting that MM may be a valuable complement to CBT in the treatment of BED.
- The proposed work builds logically from the candidate's prior work and would position her well for meeting her career goals of becoming an independent researcher who develops and evaluates mindfulness-based treatments for EWDs.
- Intervention development includes user testing (3 waves), with thematic analyses of findings. Topics for the ten CBT-MM modules have been laid out and appear to be a logical and thoughtful progression. They address common concerns for behavior change related to unhealthy eating behaviors.
- The candidate describes strong pilot data from a brief digital MM-based intervention for eating disorders designed to reduce emotional dysregulation (2-week pre-post evaluation).
- Recruitment will incorporate approaches to engage a diverse population (e.g., seeking outlets with predominantly ethnic minority populations, employing diverse images).
- Outcomes for the pilot randomized controlled trial (RCT) address a number of appropriate feasibility markers including success with recruitment/randomization, percent of eligible randomized, intervention completion, completion of assessments, and intervention acceptability. The psychological measures that will be collected include validated measures assessing a number of relevant concepts.

Weaknesses

- While the most critical feasibility outcomes are included, others with value could include adherence to recommended daily meditations, and time needed to complete study measures (a measure of participant burden).
- While the candidate explains that she has previously recruited successfully with less resources, little detail is provided about whether the proposed approach for identifying potential participants (e.g., social media ads, newspapers, podcasts, and radio stations) is one that she and/or

mentors have used in the past. No back-up plans are apparent should recruitment prove more difficult than expected. The range of options and limited proposed sample size suggest though that the approach is likely to be feasible.
- Intervention participants (both arms) will receive remote coaching by phone/text, by "trained clinical graduate students". Little information is provided about the training or monitoring of these coaches.
- The sample size for the feasibility pilot study was not justified in a rigorous statistical manner (e.g., see *Clinical and Translational Science*, 2011; Volume 4: 332–337).

4. Mentor(s), Co-Mentor(s), Consultant(s), Collaborator(s):
Strengths
- The candidate has assembled a strong mentoring team with complementary strengths, including primary mentor Dr. Evan Forman (mindfulness and other behavioral treatments for EWDs, including digital interventions) and co-mentor Dr. Hedy Kober (MM and emotional regulation). Other mentors bring strengths in areas such as health information technology/digital interventions (Dr. Bricker), career development (Dr. Feldman) and statistics/trial analysis (Dr. Aslan).

Weaknesses
- The primary mentor (Dr. Forman) is at a different institution from Dr. Sala. However, they have a history of successful remote collaboration and have outlined clear plans for weekly 1-hour remote meetings and monthly in-person meetings.

5. Environment:
Strengths
- Strong institutional support is provided from Yeshiva University, including a reduction in teaching load, should the application be awarded, to one course per year. The letter of support states that the institutional commitment to Dr. Sala is not contingent upon receipt of this K award.
- The Ferkauf Graduate School of Psychology at Yeshiva University provides substantial access to research facilities and resources. In addition, Dr. Sala has plans to visit external facilities (e.g., Dr. Bricker's laboratory and in-person meetings with Dr. Forman).

Weaknesses
- No major weaknesses noted.

Study Timeline:
Strengths
- A clear and logical timeline is laid out.

Weaknesses
- The study would not leverage a lot of existing resources such as Clinical and Translational Science Awards (CTSAs), electronic medical records (EMRs), or patient registries. However, the plan seems feasible.

Protections for Human Subjects
Acceptable Risks and Adequate Protections
- This is a low-risk study, and the application lays out the potential risks, measures for protection against risks, potential benefits, and potential importance of knowledge to be gained.

Data and Safety Monitoring Plan (Applicable for Clinical Trials Only):
- Acceptable
  - The pilot trial has a clear data and safety monitoring plan, including addressing any adverse events.

Inclusion Plans

- Sex/Gender: Distribution justified scientifically
- Race/Ethnicity: Distribution not justified scientifically
- For NIH-Defined Phase III trials, Plans for valid design and analysis: Not applicable
- Inclusion/Exclusion Based on Age: Distribution justified scientifically
- Older adults are excluded to reduce sample heterogeneity for intervention development.

Vertebrate Animals
Not Applicable (No Vertebrate Animals)

Biohazards
Not Applicable (No Biohazards)

Resubmission
- This resubmission application appears to be very responsive to prior comments (e.g., mentoring team adjusted and justified to increase clinical trial and statistical expertise, institution now would reduce the teaching load, and innovation aspect enhanced with a focus on digital intervention).

Training in the Responsible Conduct of Research
Acceptable
Comments on Format:
- The format includes a combination of face-to-face and online instruction.

Comments on Subject Matter:
- The required range of subjects is addressed.

Comments on Faculty Participation:
- Mentor roles are specified.

Comments on Duration:
- A sufficient duration is proposed.

Comments on Frequency:
- A sufficient frequency if proposed.

Resource Sharing Plans
Acceptable
- The plan will adhere to the National Institutes of Health (NIH) Grants Policy on Sharing of Unique Research Resources.

Budget and Period of Support
Recommend as Requested

CRITIQUE 4

Overall Impact: This is a K23 resubmission application by an impressive early career-stage investigator. Dr. Sala has a record of 39 peer-reviewed publications, has obtained prior research funding including a National Science Federation Graduate Research Fellowship, and has demonstrated a strong commitment to mindfulness and eating behavior research. She has assembled an excellent mentorship team, and she addressed the prior reviewers' concerns quite well. For example, she improved the mentorship team by adding mentors with unique expertise to minimize overlap (e.g., qualitative methods, biostatistics, technology-based interventions), added more structured trainings, incorporated stakeholders in the development of the intervention, and demonstrated stronger institutional commitment through a reduced teaching load. There is a strong scientific rationale for the combination of cognitive behavioral therapy (CBT) and mindfulness meditation (MM) to target both

emotion dysregulation and dietary over-restriction as core mechanisms underlying binge eating disorder (BED). Dr. Sala and co-mentor Dr. Kober recently developed and found preliminary support for the acceptability and feasibility of a brief digital mindfulness intervention for eating disorders, which provides strong preliminary data to support the proposed work. The K23 application research plan includes iterative digital intervention development with user input, followed by a pilot randomized controlled trial (RCT). The candidate proposes to use software programs for digital treatment development that her mentor has previously used, enhancing feasibility of the plan. The intervention content is well described and will be revised based on user input. The pilot RCT includes digital CBT app-based treatment for BED as an appropriate control condition, with similar amount and length of treatment content. The pilot trial includes clearly defined acceptability/feasibility benchmarks (although benchmarks of 6.5/10 for perceived usability and helpfulness seem relatively low). Weaknesses in the approach include: 1) although the candidate and co-mentor successfully conducted pilot data virtually, it is unclear whether they have conducted national recruitment as proposed; 2) more detail is needed on the text messages to be sent as part of the intervention; and 3) while the rationale for asking digital CBT participants not to meditate is to prevent contamination is logical, those instructions could have an unintended reverse effect (and contamination is not likely to be an issue given the national recruitment strategies). These weaknesses are viewed as minor given this highly significant application and excellent candidate with strong promise for independent research on mindfulness-based treatments for eating and weight disorders.

THE FOLLOWING SECTIONS WERE PREPARED BY THE SCIENTIFIC REVIEW OFFICER TO SUMMARIZE THE OUTCOME OF DISCUSSIONS OF THE REVIEW COMMITTEE, OR REVIEWERS' WRITTEN CRITIQUES, ON THE FOLLOWING ISSUES:

PROTECTION OF HUMAN SUBJECTS: ACCEPTABLE

INCLUSION OF WOMEN PLAN: ACCEPTABLE

INCLUSION OF MINORITIES PLAN: ACCEPTABLE

INCLUSION ACROSS THE LIFESPAN: ACCEPTABLE

TRAINING IN THE RESPONSIBLE CONDUCT OF RESEARCH: ACCEPTABLE

COMMITTEE BUDGET RECOMMENDATIONS: The budget was recommended as requested.

Footnotes for 1 K23 AT012126-01A1; PI Name: Sala, Margarita

NIH has modified its policy regarding the receipt of resubmissions (amended applications).See Guide Notice NOT-OD-18-197 at https://grants.nih.gov/grants/guide/notice-files/NOT-OD-18-197.html. The impact/priority score is calculated after discussion of an application by averaging the overall scores (1-9) given by all voting reviewers on the committee and multiplying by 10. The criterion scores are submitted prior to the meeting by the individual reviewers assigned to an application, and are not discussed specifically at the review meeting or calculated into the overall impact score. Some applications also receive a percentile ranking. For details on the review process, see http://grants.nih.gov/grants/peer_review_process.htm#scoring.

MEETING ROSTER

The roster for this review meeting is displayed as an aggregated roster that includes reviewers from multiple AT Special Emphasis Panels Meetings
for the 2023/01 council round.
This roster for AT is available here.
